# Development of Glycyrrhetinic Acid and Folate Modified Cantharidin Loaded Solid Lipid Nanoparticles for Targeting Hepatocellular Carcinoma

**DOI:** 10.3390/molecules27206786

**Published:** 2022-10-11

**Authors:** Yilin Xu, Min Wang, Shuangcheng Ning, Zhonglan Yang, Lili Zhou, Xinhua Xia

**Affiliations:** 1 School of Pharmacy, Hunan University of Chinese Medicine, Changsha 410208, China; 2 The Second Hospital of Hunan University of Chinese Medicine, Changsha 410005, China

**Keywords:** cantharidin, solid lipid nanoparticles, glycyrrhetinic acid, folate, antitumor, liver-targeting

## Abstract

Cantharidin (CTD) is the major component of anticancer drugs obtained from *Mylabris Cichorii* and has a good inhibitory effect on several cancers, including hepatocellular carcinoma (HCC) and breast cancer. However, due to its toxicity, oral administration can cause various adverse reactions, limiting its clinical application. The aim of this work was to design glycyrrhetinic acid (GA)- and/or folate (FA)-modified solid lipid nanoparticles (SLNs) for the encapsulation of CTD to target HCC. Four CTD-loaded SLNs (cantharidin solid lipid nanoparticles (CSLNs), glycyrrhetinic acid-modified cantharidin solid lipid nanoparticles (GA-CSLNs), folate-modified cantharidin solid lipid nanoparticles (FA-CSLNs), and glycyrrhetinic acid and folate-modified cantharidin solid lipid nanoparticles (GA-FA-CSLNs)) were prepared by the emulsion ultrasonic dispersion method, and their physicochemical parameters were determined (particle size and distribution, morphology, zeta-potential, entrapment efficiency, drug loading, and hemolysis). Additionally, the antitumor activities of the four SLNs were evaluated comprehensively by tests for cytotoxicity, cell migration, cell cycle, apoptosis, cellular uptake, competition suppression assay, and in vivo tumor suppression assay. Four SLNs showed spherical shapes and mean diameters in the range of 75–110 nm with size dispersion (PDI) within the range of 0.19–0.50 and zeta-potential approximately –10 mV. The entrapment efficiency of CTD in SLNs was higher than 95% for all tested formulations, and no hemolysis was observed. Compared to GA-CSLNs or CSLNs, GA-FA-CSLNs and FA-CSLNs showed stronger cytotoxicity on hepatocellular carcinoma cells (HepG2), and the cytotoxicity of GA-FA-CSLNs on hepatocyte cells (L-02) was remarkably reduced compared with other formulations. GA-FA-CSLNs and FA-CSLNs also increased the inhibition of HepG2 cell migration, and FA-CSLNs had the highest apoptosis rate. The cell cycle results indicated that HepG2 cells were arrested mainly in the S phase and G2/M phase. Analysis of competition inhibition experiments showed that GA and FA ligands had targeted effects on HepG2 cells. The in vivo tumor inhibition experiment showed that GA-FA-CSLNs and FA-CSLNs had excellent tumor inhibition ability—their tumor inhibition rates were 96.46% and 89.92%, respectively. Our results indicate that GA-FA-CSLNs and FA-CSLNs have a promising future in the therapeutic intervention of HCC.

## 1. Introduction

Hepatocellular carcinoma (HCC) is the most common form of primary liver cancer and usually occurs in the context of chronic liver disease [1,2]. Currently, in the treatment of HCC, great progress has been made in surgical resection, liver transplantation, immunotherapy, chemotherapy, local treatment, including radiofrequency ablation and microwave ablation, etc. [3,4,5,6,7,8]. The disease prognosis is usually poor because most patients with HCC are diagnosed at an advanced stage of disease and because of a lack of accuracy and individualization in the selection and application of treatment modes; a further influencing factor is the high invasiveness of HCC itself [9]. In recent years, targeted therapies have been clinically used to treat a variety of cancers, including liver cancer [10,11]. The development of new Chinese herbal dosage forms that can target liver cancer cells may provide better synergistic therapy for HCC patients.

Cantharidin (CTD) is an effective antitumor component in the Chinese traditional medicine cantharides [12,13]. It has demonstrated a good therapeutic effect on malignant tumors, especially in advanced cancers, including gastric cancer [14], liver cancer [15,16], breast cancer [17], lung cancer [18], oral squamous cell carcinomas [19], etc. [20,21]. Its pertinent anticancer mechanisms on cancerous cells include protein phosphatase 1 and protein phosphatase 2A inhibition, apoptosis induction, and protein synthesis alteration [22]. However, the disadvantages of CTD have greatly limited its application in clinical therapy [23,24]. On the one hand, CTD has a short biological half-life and poor stability. On the other hand, it has been reported that CTD has high hepatotoxicity [25] and causes irritation, inflammation, sinus tachycardia, and even death in severe cases [26]. Therefore, researchers have attempted to modify CTD through preparative techniques to improve its liver-targeting ability, concentrate it in the liver, improve its efficacy, and reduce drug concentrations in other tissues.

Solid lipid nanoparticles (SLNs) are a kind of solid-core lipid nanocarrier that can hold both hydrophilic and hydrophobic drugs; they can be composed of biocompatible ingredients [27]. Drug-loaded solid colloidal delivery systems have become a research hotspot due to their advantages of controlled release, avoiding drug leakage, low toxicity, and good biocompatibility [28]. In addition, previous studies have shown that SLNs can improve the bioavailability of drugs by altering the dissolution of drugs and can be used to improve the tissue distribution of drugs [29,30]. Thus, SLNs coloaded with CTD could be used to efficiently enrich drugs in tumor tissue, reduce the drug dose, and prevent damage to normal organs or tissues, and they have the potential for targeted therapy in tumors. Furthermore, the surface modification of SLNs can improve their unique capability to target specific receptors [31]. Therefore, SLNs are one of the first choices for drug delivery.

There are many specific binding sites on the liver cancer cell membrane that can bind to GA, and the binding of GA to these sites is saturable and highly specific [32,33]. Folate receptor (FR) is a glycosylated phosphatidylinositol-linked transmembrane single-chain glycoprotein. As cancer progresses, the density of FR on the surface of tumor cells increases [34,35]. The literature shows that FR is highly expressed in primary liver cancer tissues and cells [36,37,38]. The high affinity between FA and FR can be used to treat primary liver cancer with targeted agents modified by FA, which can greatly improve the curative effect and reduce side effects [39]. The aim of this study was to design CTD-loaded SLNs modified by FA and/or GA in order to enhance the antihepatocellular-carcinoma effect of CTD and reduce its toxicity.

## 2. Results and Discussion

### 2.1. Characterization of SLNs

**Figure 1:**
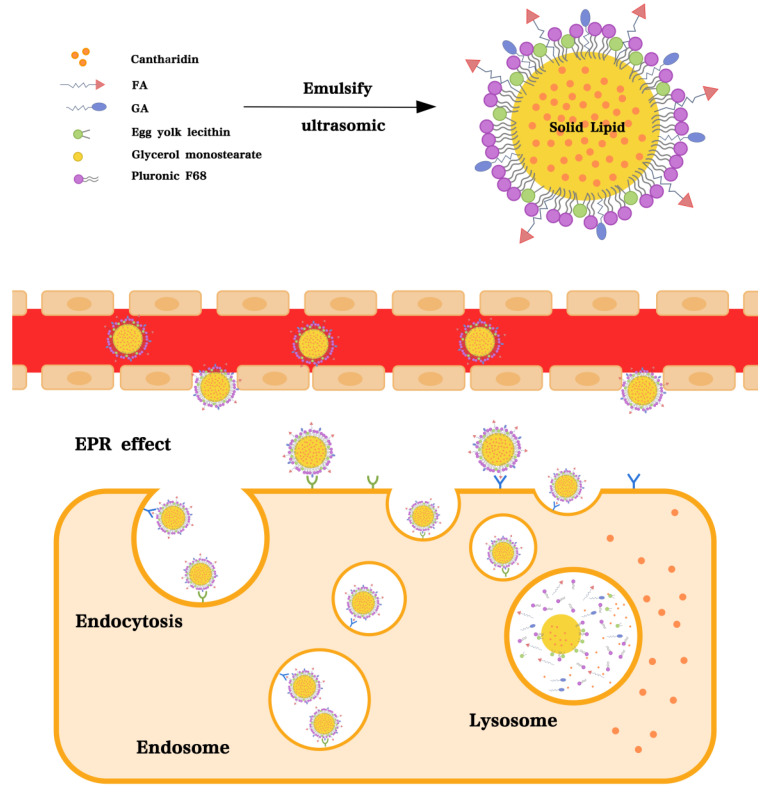
Schematic diagram of GA-FA-CSLNs.

**Figure 2:**
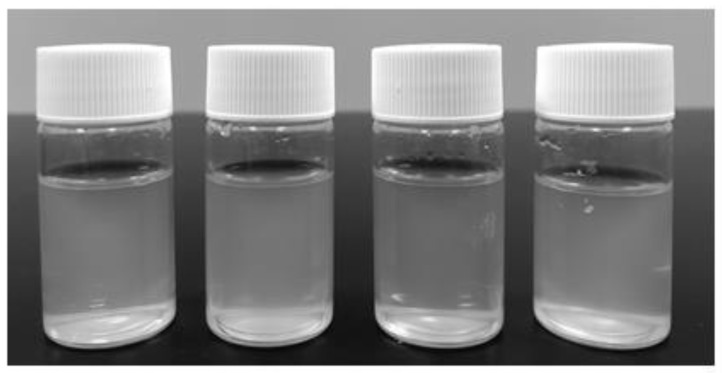
Appearance of SLNs (CSLNs, GA-CSLNs, FA-CSLNs, and GA-FA-CSLNs in order from left to right).

**Figure 3:**
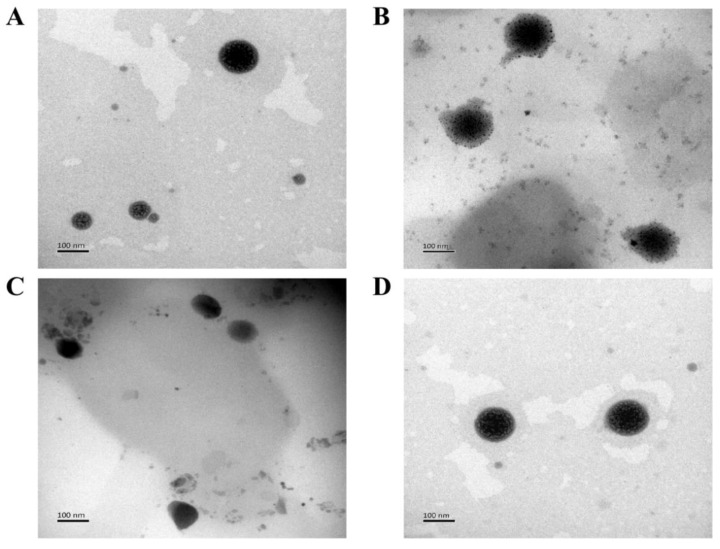
Transmission electron micrograph (**A**) CSLNs; (**B**) GA-CSLNs; (**C**) FA-CSLNs; and (**D**) GA-FA-CSLNs.

Four kinds of CTD-loaded SLNs (CSLNs, GA-CSLNs, FA-CSLNs, and GA-FA-CSLNs) were successfully prepared by emulsion ultrasonic dispersion (Figure 1). Macroscopic observations revealed that the SLN dispersions obtained were clear and transparent, accompanied by light blue opalescence, as shown in Figure 2. For antitumor treatment, nanoparticle size is crucial. If the particles are too small, they will be quickly eliminated by the kidneys, whereas if the size surpasses 300 nm, the reticuloendothelial system (RES) readily captures and traps them, making it difficult for them to reach the target site. To evaluate nanoparticle morphology, we characterized the prepared four kinds of CTD-loaded SLNs with transmission electron microscopy (TEM) and dynamic light scattering (DLS). Microstructure analysis showed that SLNs were relatively round, without adhesion between particles, according to the TEM image in Figure 3. Therefore, the addition of targeted molecules may help to prevent particle aggregation. The particle size, polydispersity index (PDI), zeta-potential (ZP), encapsulation efficiency (EE%), and drug loading (DL) of SLNs are presented in Table 1. The findings showed that the sizes of four SLNs were adequate for the prevention of renal leakage and RES clearance. Notably, the particle size of the FA-CSLNs and GA-FA-CSLNs was similar, indicating that the size of the SLNs did not significantly increase after modification. The encapsulation rate of ligand-modified CSLNs was greater than 95% and the drug loading was above 0.30%, indicating that the encapsulation rate and drug loading of SLNs were not affected by targeted molecular modification. The zeta-potential results indicated that modified CSLNs exhibited better potential results than CSLNs, indicating that the addition of targeted molecules may help to improve CSLNs stability.

### 2.2. Hemolysis Test

SLN formulations have been attracting increasing attention for intravenous application [40]. In vitro hemolysis tests can be used as a screening method for the hemolytic toxicity of intravenous preparations and are a significant indicator of the safety of preparations [41]. In this work, we investigated the hemocompatibility of nanoparticles by examining the hemolysis of CSLNs, GA-CSLNs, FA-CSLNs, and GA-FA-CSLNs at different concentrations to prepare for subsequent intravenous injection.

**Figure 4:**
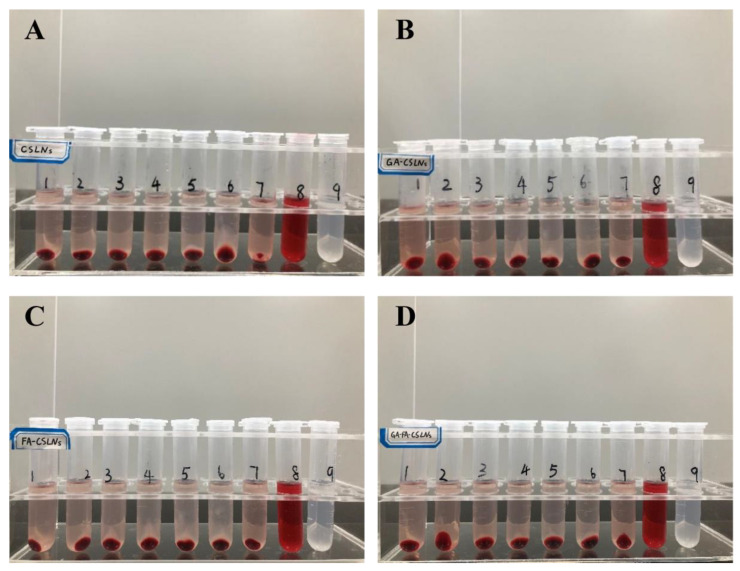
Hemolysis assessment of SLNs (**A**) CSLNs; (**B**) GA-CSLNs; (**C**) FA-CSLNs; and (**D**) GA-FA-CSLNs.

As shown in Figure 4, macroscopic observations showed no significant differences between tubes 1–6 and tube 7 of the four CTD-loaded SLNs (CSLNs, GA-CSLNs, FA-CSLNs, and GA-FA-CSLNs), proving that the solid lipid nanoparticles had good biocompatibility in the experimental concentration range and met the requirements for intravenous injection.

### 2.3. Cell Proliferation Inhibition Assay

**Figure 5:**
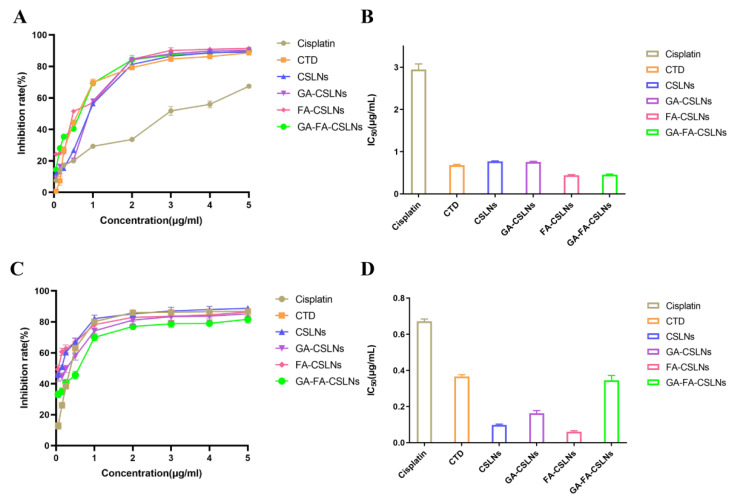
In vitro cytotoxicity of SLNs on HepG2 cells and L-02 cells; (**A**) the inhibition rate of HepG2 cells; (**B**) the values of HepG2 cells IC_50_; (**C**) the inhibition rate of L-02 cells; (**D**) the values of L-02 cells IC_50_. Date is presented as the mean ± SD (*n* = 6).

The in vitro cytotoxicities of SLNs modified with various ligands in HepG2 and L-02 cells are shown in Figure 5. For HepG2 cells, all cantharidin groups had higher inhibitory rates than cisplatin (the positive control group); furthermore, the cell inhibition rate remarkably increased in a dose-dependent fashion within the concentration range of 0.05–1 μg/mL, and the inhibition rates of the four SLNs (CSLNs, GA-CSLNs, FA-CSLNs, and GA-FA-CSLNs) were higher than those of the CTD solutions when the concentration was greater than 2μg/mL; this was especially true for FA-CSLNs, indicating that the surface FA modification contributed to the selective cytotoxicity of the nanoparticles via FA-receptor-mediated intracellular delivery. Therefore, CSLNs modified by FA had a stronger inhibitory effect on HepG2 liver cancer cells.

The IC_50_ values of the four SLNs to HepG2 and L-02 cells are summarized in Figure 5. The IC_50_ values for L-02 cells were much lower than those for HepG2 cells. In addition, the IC_50_ values of the four SLNs were lower than those of the CTD solution group, indicating that the nanoparticles could be better absorbed by cells. Results indicated that both CSLNs and modified CSLNs effectively inhibited the proliferation of HepG2 and L-02 cells, and the inhibitory effect of GA-FA-CSLNs on HepG2 cells was stronger than that of other SLNs.

### 2.4. Cell Migration Assays

**Figure 6:**
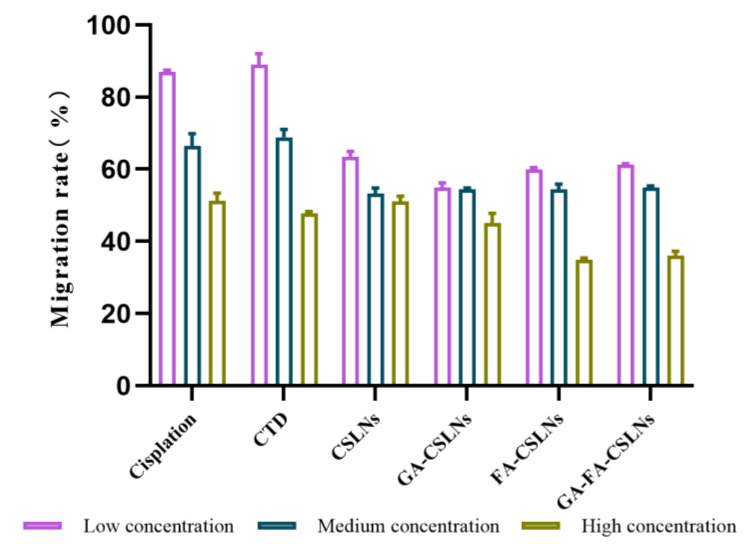
Migration rate of HepG2 cells (mean ± SD, *n* = 3).

**Figure 7:**
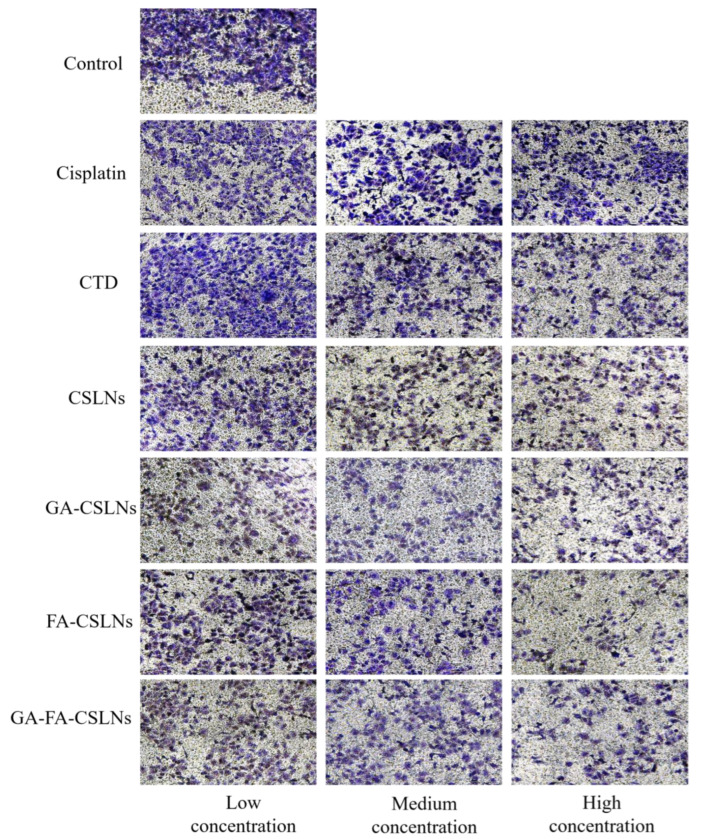
Cell migration assays at 24 h after treatment with various formulations observed at 10 × 10 magnification.

The transwell assay was used to evaluate the effects of the four CTD-loaded SLNs (CSLNs, GA-CSLNs, FA-CSLNs, and GA-FA-CSLNs) on the migration of HepG2 cells. As shown in Figure 6 and Figure 7, massive cells migrated to the lower chamber in the control and CTD groups, whereas the number of migrated cells in the four CTD-loaded SLN groups decreased significantly. Notably, consistent with the cell proliferation inhibition results, similar migration rates were noted between the high-dose FA-CSLNs group and the GA-FA-CSLNs group, whereas the state of migration in the CSLNs group was worse.

The results revealed that, compared with the CTD solution and cisplatin group, the four CTD-loaded SLNs had a stronger inhibitory effect on the migration of HepG2 cells that was dose-dependent.

### 2.5. Apoptosis Assay

**Figure 8:**
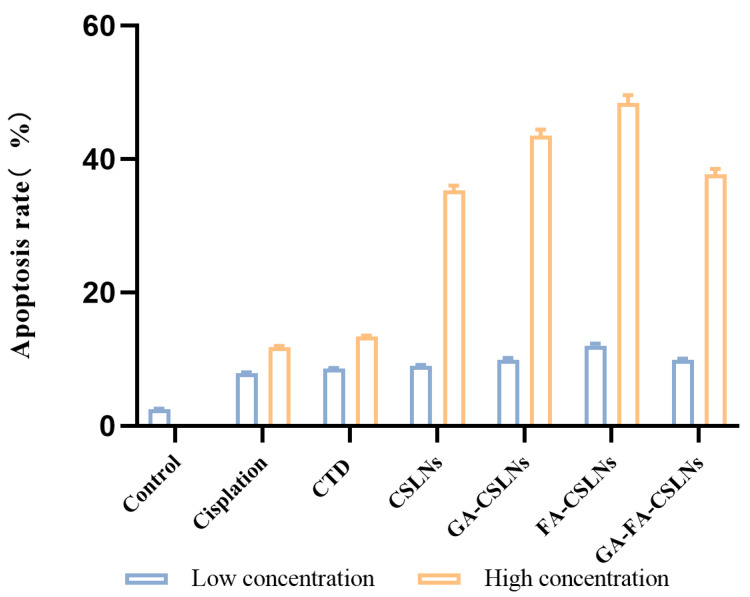
Apoptosis rate of HepG2 cells (mean ± SD, *n* = 3).

**Figure 9:**
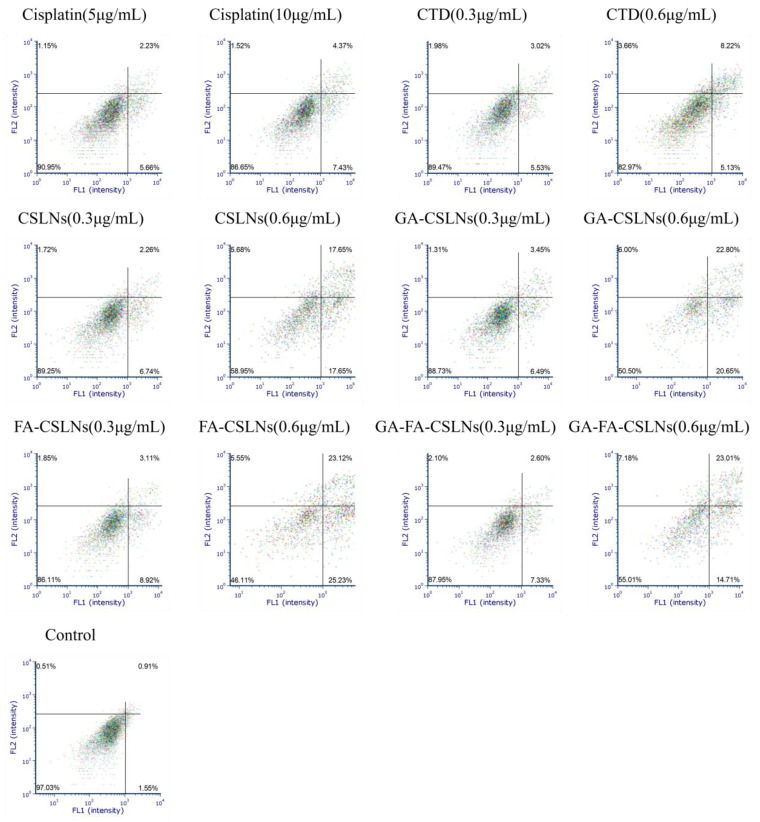
Apoptosis of HepG2 cells with CSLNs modified by different ligands.

In this study, flow cytometry was used to detect the effect of the four CTD-loaded SLNs (CSLNs, GA-CSLNs, FA-CSLNs, and GA-FA-CSLNs) on the apoptosis of HepG2 cells, as shown in Figure 8 and Figure 9. Compared to the blank control, the apoptosis rate of all drug groups was slightly increased (approximately 10%) at low concentrations, while at high concentrations, the cell apoptosis rates of the four CTD-loaded SLNs were significantly higher than those of the CTD solution and cisplatin group at high concentrations. Treatment at 0.6 µg/mL induced a further increment of early apoptotic, late apoptotic, and early necrotic cell populations, suggesting that the fraction of apoptotic cells varied with the SLN concentration in a dose-dependent manner. Moreover, at the same concentration, the apoptosis rate of HepG2 cells treated with FA-CSLNs or GA-CSLNs was higher than that of those treated with GA-FA-CSLNs, which may be because GA could trigger protective autophagy in HepG2 cells [42]. In conclusion, an increased dose of drug-loaded SLNs resulted in a substantial increment in late apoptotic/early necrotic cell populations.

The above results indicate that the SLNs were highly absorbed by HepG2 cells and improved the apoptotic activity of the HepG2 cells.

### 2.6. Cell Cycle Evaluation

**Figure 10:**
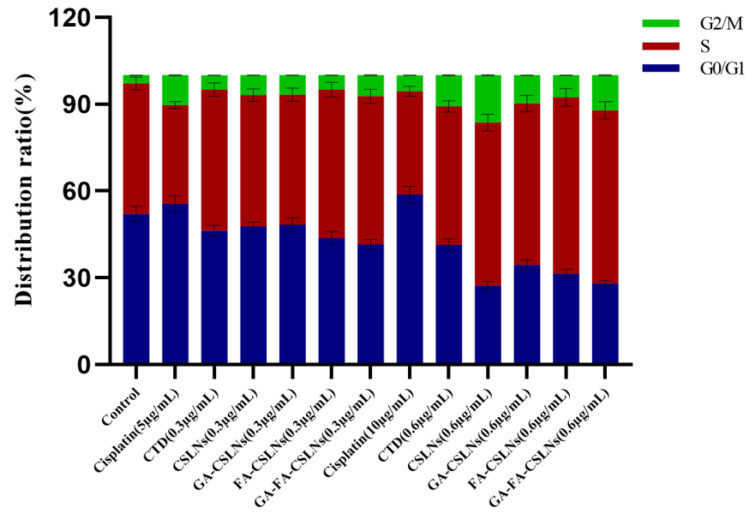
Cell-cycle distribution of HepG2 cells (mean ± SD, *n* = 3).

**Figure 11:**
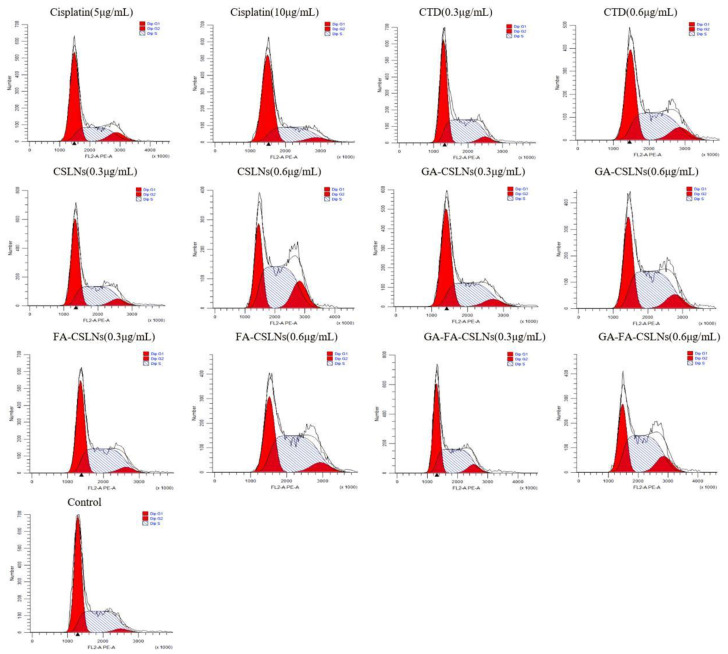
Cell cycle diagram of SLNs modified with different ligands on HepG2.

Next, we examined the effects of the four CTD-loaded SLNs (CSLNs, GA-CSLNs, FA-CSLNs, and GA-FA-CSLNs) on the cell cycle using flow cytometry, as shown in Figure 10 and Figure 11. For the CTD solution group, the percentage of cells in the G2/M phase increased compared with the blank control, suggesting cell cycle arrest in the G2/M phase. For the four CSLNs, the percentage of cells in the S and G2/M phases increased significantly compared with the blank control in a dose-dependent manner, indicating that the cell cycle was mainly arrested in the S and G2/M phases. Among them, CSLNs mainly blocked the cell cycle in the G2/M phase while GA-CSLNs, FA-CSLNs, and GA-FA-CSLNs mainly blocked the cell cycle in the S phase, suggesting that the CSLNs modified by the GA and/or FA ligand can inhibit DNA synthesis and promote cell apoptosis. For the cisplatin group, cell cycle arrest was in the G2/M phase at low concentrations and in the G0/G1 phase at high concentrations.

### 2.7. Cellular Uptake and Competition Suppression Assay

**Figure 12:**
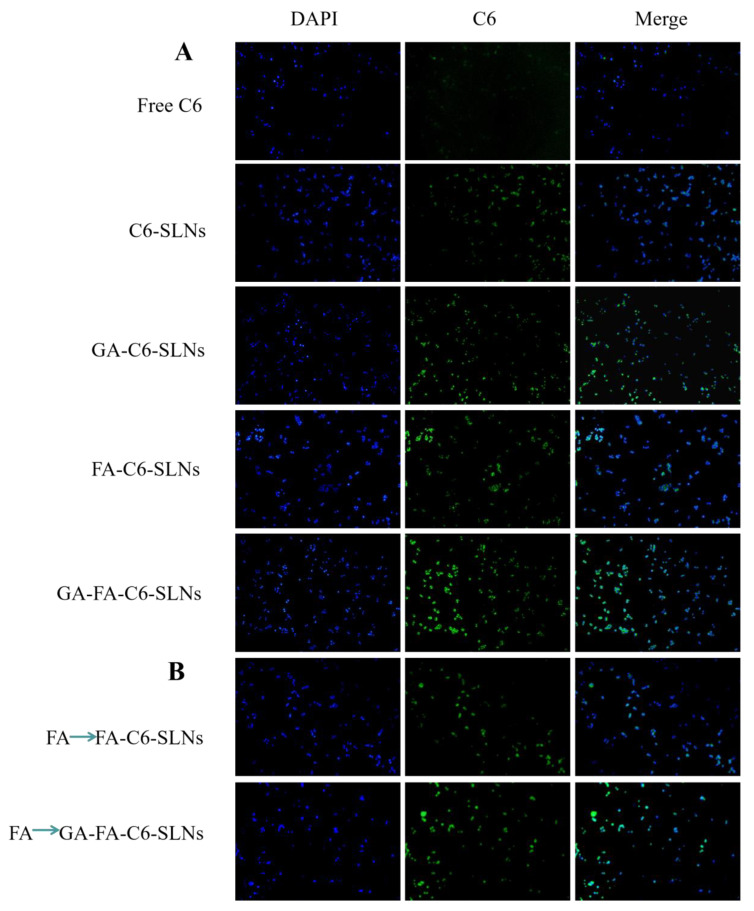
Uptake of HepG2 cells by C6-SLNs modified with different ligands. (**A**) Cellular uptake; (**B**) competition suppression assay.

In this study, CTD was replaced by using the fluorescent dye C6 in the four SLNs to prepare C6-loaded SLN formulations. HepG2 cells were chosen as a cell model. An inverted fluorescence microscope was used to observe the cellular uptake behaviors of the samples, and green fluorescence in the cytoplasmic region indicated an efficient uptake of C6-containing samples by cells. For HepG2 cells, the intracellular fluorescence was observed in cells treated with the four C6-loaded SLNs, indicating that all the above samples could be ingested by HepG2 cells (Figure 12A). Furthermore, the fluorescence intensity of C6 loaded in SLNs was always stronger than that of C6 not loaded in SLNs. This might be because the SLNs are more easily taken up by the cells via endocytosis.

Interestingly, for the GA-FA-C6-SLNs group, by contrast, intensified fluorescence was observed inside the cells. This enhanced cellular uptake could be ascribed to the GA- and FA-mediated assimilation via glycyrrhetinic acid receptor and folate receptor binding, respectively. To verify this, we pretreated the cells with free FA to saturate the folate receptors. In this case, the intracellular fluorescence was strongly weakened in the FA-C6-CSLNs group, further confirming the critical contribution of folate-receptor-mediated internalization (Figure 12B). Notably, stronger fluorescence was observed in the GA-FA-C6-CSLNs group, indicating that the GA-mediated assimilation could occur via glycyrrhetinic acid receptor binding after the folate receptors were saturated, confirming the contribution of glycyrrhetinic acid-receptor-mediated internalization and further increasing the uptake of the contents. These findings suggest that the modification of targeted molecules promotes the cellular uptake of the contents.

### 2.8. In Vivo Efficacy Evaluation

**Figure 13:**
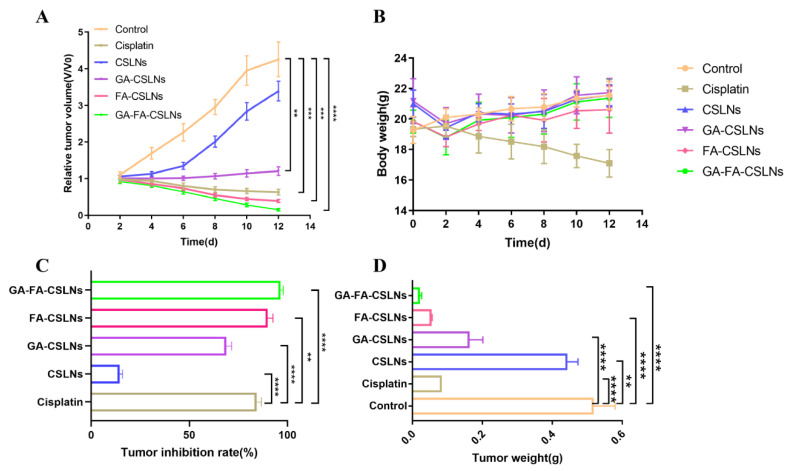
In vivo pharmacodynamic evaluation index of CSLNs (**A**) relative tumor volume; (**B**) body weight; (**C**) tumor inhibition ratio; (**D**) tumor weight. Date is presented as the mean ± SD (*n* = 6). ** *p <* 0.01, *** *p <* 0.001, and **** *p <* 0.0001.

**Figure 14:**
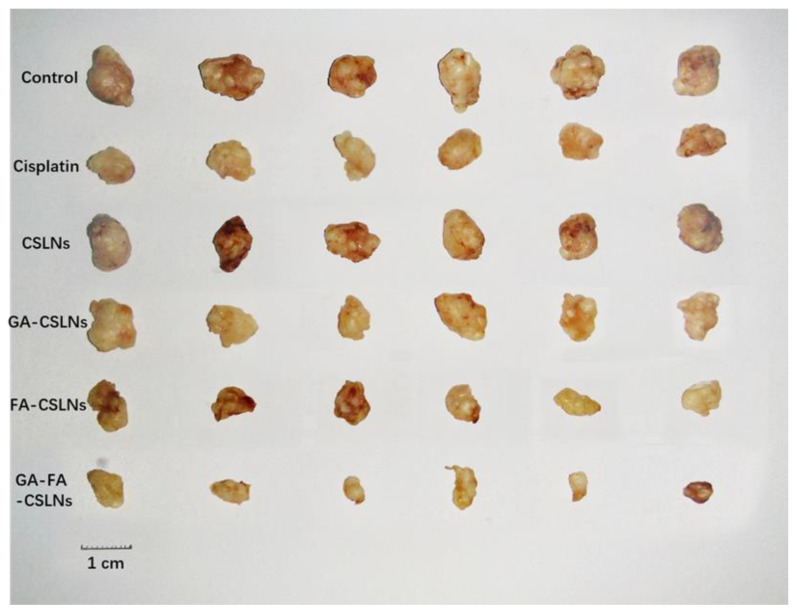
CSLNs modified with different ligands on tumor-bearing nude mice after dissection of tumor (*n* = 6).

To assess the antitumor potential of the four SLNs, nude mice bearing subcutaneous xenograft tumors were used for in vivo tumor inhibition experiments. As shown in Figure 13A,C,D, and Figure 14, the dynamic efficacy was monitored by measuring the tumor size. The inhibitory effects of the four SLNs on liver tumors are as follows: GA-FA-CSLNs > FA-CSLNs > GA-CSLNs > CSLNs. Among these, GA-FA-CSLNs and FA-CSLNs showed excellent tumor inhibition, and their tumor inhibition rates were 96.46% and 89.92%, respectively—better than that of cisplatin (84.31%). With FA modification, the tumor suppression effect was enhanced to some extent for FA-CSLNs, which can be attributed to the active tumor target. Among them, the best efficacy was achieved for GA-FA-CSLNs, with almost complete tumor growth inhibition, demonstrating that CTD has an excellent antitumor effect in vivo after being modified by these two targeted molecules. The reason for this is that GA-mediated and FA-mediated assimilation could occur via glycyrrhetinic acid receptor and folate receptor binding, promoting the tumor uptake of the GA-FA-CSLNs. In addition, no significant changes in body weight were observed in mice treated with modified SLNs, indicating that they were well-tolerated at the tested dose (Figure 13B). The above results suggest that GA-FA-CSLNs and FA-CSLNs have a good clinical application prospect in antiliver-tumor therapy.

## 3. Materials

### 3.1. Reagents

Cantharidin was obtained from Xi’an Tongze Biotechnology Co., Ltd. (Xi’an, China). GA was synthesized in our Laboratory (Hunan University of Chinese Medicine, China). FA was obtained from Shanghai Pengshuo Biotechnology Co., Ltd. (Shanghai, China). Egg yolk lecithin was supplied by Lipoid Brand (Köln, Germany). Glycerol monostearate was received from Hengxin Chemical Reagent Co., Ltd. (Shanghai, China). Pluronic F-68 was purchased from Source Leaf Biology Co., Ltd. (Shanghai, China). Dialysis bag (MWCO 8000–14,000 Da) was bought from Beijing Dingguo Changsheng Biotechnology Co., Ltd. (Beijing, China). MTT was obtained from Biosharp (Hefei, China). Annexin V-FITC/PI Apoptosis Detection Kit was purchased from Becton, Dickinson and Company (Franklin Lakes, NJ, USA).

### 3.2. Cell Lines

HepG2 cells were purchased from West China School of Pharmacy, Sichuan University (Sichuan, China), and L-02 cells were purchased from Procell Life Science&Technology Co., Ltd. (Wuhan, China). HepG2 cells were cultured in glucose DMEM medium (10% FBS; 0.75% penicillin-streptomycin) with 37 °C/5% CO_2_. The L-02 cells were cultured in RPMI-1640 medium (10% FBS; 0.75% penicillin-streptomycin) in an incubator containing 5% CO_2_ at 37 °C.

### 3.3. Animals

Forty BALB/c nude mice (4–6 weeks old, half male and half female) were purchased from Hunan STA Laboratory Animal CO., Ltd. (Changsha, China) and raised in Laboratory Animal Center, Hunan University of Chinese Medicine. These animals were kept in a sterile environment and given free access to water and food. Experiments were performed in compliance with the requirements of the animal ethics committee of Hunan University of Chinese Medicine. (ethical accreditation No. LL2019100805).

## 4. Methods

### 4.1. Preparation of Four CTD-Loaded SLNs

Four CTD-loaded SLNs (CSLNs, GA-CSLNs, FA-CSLNs, and GA-FA-CSLNs) were prepared by the emulsion ultrasonic dispersion method. For CSLNs, 25 mg of glycerol monostearate, 50 mg of egg yolk lecithin, and 2 mg of CTD were dissolved in 5 mL of methanol to form an organic phase and kept warm in a 60 °C water bath, then a hot aqueous solution (15 mL) containing 2.0% (*w/v*) of Pluronic F-68 was slowly injected into the organic phase under magnetic stirring (1000 r/min), followed by continuous stirring for 1 h to remove methanol. Subsequently, the resulting CSLNs were collected after sonication for 30 min at 20% power with a probe sonicator. Finally, the obtained CSLNs were diluted to 20 mL and filtered through a 0.22 µm microporous membrane, and filtrate was collected for reserve. For the CSLNs modified by targeted ligands, including GA-CSLNs, FA-CSLNs, and GA-FA-CSLNs, 10 mg of GA and/or FA ligands were added to the organic phase during preparation, and the remaining operations were the same as above.

### 4.2. Characterization

The physicochemical properties of the four CTD-loaded SLNs (CSLNs, GA-CSLNs, FA-CSLNs, and GA-FA-CSLNs) were characterized. Particle size, polydispersity index (PDI), and zeta-potential were measured with a Zetasizer Nano ZS90 analyzer (Malvern Instruments, Great Malvern, UK). The surface morphology of the SLNs was analyzed with a transmission electron microscopy (TEM).

Encapsulation efficiency (EE%) refers to the ratio of drug encapsulated in SLNs to the total amount of CTD (Mtotal) in the SLNs preparation. The EE% was determined using a dialysis method. The appropriate amount of methanol–acetonitrile mixture (at a ratio of 1:1, *v*/*v*) was added to 1 mL of SLN to determine the Mtotal. Approximately 5 mL of CSLNs was added to a dialysis bag, and then the dialysate was concentrated to 2 mL to determine the Mfree in SLNs. Both Mtotal and Mfree were analyzed by HPLC (1260 Infinity II, Agilent Technologies Inc., Santa Clara, CA, USA) and calculated with the following equation:

EE(%)=(Mtotal−Mfree)/Mtotal×100%

where Mtotal is the total amount of CTD in SLNs, and Mfree is the amount of CTD remaining in the solution.

Drug loading (DL) refers the ratio of total amount of drug (Mtotal) in the SLNs to the weight of SLNs themselves (MSLNs). To calculate MSLNs, 2 mL of CSLNs was added to a weighing bottle and freeze-dried for 48 h. The freeze-dried powders were treated with the same method as in the determination of encapsulation efficiency to measure Mtotal, then DL was calculated according to the following equation:

DL(%)=(Mtotal/MSLNs)×100%

where Mtotal is the weight of CTD in the freeze-dried powder, and MSLNs is the weight of freeze-dried powder.

### 4.3. Hemolysis Test

Healthy rabbit blood was stirred to remove fibrinogen; the fibrinogen-free blood was washed 3 times with isotonic 0.9% NaCl solution and centrifuged at 1000 rpm for 15 min each time. The supernatant was discarded, and the obtained erythrocytes were resuspended in 0.9% NaCl solution and stored at 4℃.

The hemolysis test was carried out according to standard [43]; numbers 1–6 were the test tubes, number 7 was the negative control tube, number 8 was the positive control tube, and number 9 was the sample control tube. The experimental scheme is shown in Table 2. Samples were then incubated in a thermostat at 37 °C for 3 h to observe hemolysis and coagulation.

### 4.4. Cell Proliferation Inhibition Assay

The effect of CSLNs on the survival rate of HepG2 and L-02 cells was detected by MTT colorimetry. The HepG2 cells (7000 cells/well) or L-02 cells (10,000 cells/well) were inoculated in 96-well plates and incubated at 37 °C for 16 h, and then treated with CSLNs of different CTD concentrations (0, 0.05, 0.15, 0.25, 0.5, 1, 2, 3, 4, and 5 µg/mL) for 72 h. Then, cells were washed with PBS twice and 100µL MTT (0.5 mg/mL) was added to each well. Afterwards, the cells were further incubated at 37 °C for 4 h. Finally, 150 µL of DMSO was added into each well and the plates were shaken in a horizontal oscillator for 15 min in the dark. The absorbance of cell viability was measured with an ELISA Microplate Reader (ELx800), and half inhibitory concentration (IC_50_) values were calculated by IBM SPSS Statistics 21.0 (IBM Corp. Armonk, NY, USA).

Cisplatin [44], CTD solution, GA-CSLNs, FA-CSLNs, and GA-FA-CSLNs were measured with the same method as CSLNs.

### 4.5. Cell Migration Assays

Transwell assay was used to evaluate cell migration. Briefly, 100 μL of HepG2 cell suspension (approximately 1 × 10^6^ cells/mL) without serum medium was added into the inner chamber and incubated for 1 h. Then, 1 mL DMEM high glucose with 10% FBS was added to the bottom of the transwell. After the cells adhered to the wall, three concentrations (0.3, 0.6, 0.9 μg/mL) of CTD solution and the four SLNs (CSLNs, GA-CSLNs, FA-CSLNs, and GA-FA-CSLNs) were used separately to incubate cells followed by culture for 24 h. In addition, three concentrations (5, 10, 15 μg/mL) of cisplatin were used as the positive control group. Then, the medium was removed, the cells were washed twice with PBS, the traversed cells were fixed with paraformaldehyde solution for 20 min and then stained with crystal violet for 30 min. Finally, cells were observed under 10× power inverted fluorescence microscope (DMILLED) and the migration rates were calculated.

### 4.6. Apoptosis Assay

HepG2 cells (1 × 10^6^/mL) were added into 12-well cell culture plates; two concentrations (0.3, 0.6 μg/mL) of CTD solution and the four SLNs (CSLNs, GA-CSLNs, FA-CSLNs, and GA-FA-CSLNs) were separately added to the culture bottles, and then the bottles were cultured for 48 h. Two concentrations (5, 10 μg/mL) of cisplatin were used as the positive control group. Administration was terminated and cells in suspension were washed away with PBS. Afterwards, the cells were digested with trypsin without EDTA for 4 min, centrifuged at 1000 rpm for 3 min, and washed with PBS twice. Subsequently, cells were resuspended with 100 μL of buffer and each tube was added with 5 μL of Annexin V-FITC antibody and 5 μL of PI antibody buffer, followed by staining for 15 min in the dark. Finally, 200 μL of 1 × Buffer was added and measured by flow cytometry (CytoFLEX).

### 4.7. Cell-Cycle Detection

HepG2 cells (1 × 10^6^/mL) were added into 12-well cell culture plates with CTD solution and SLNs (CSLNs, GA-CSLNs, FA-CSLNs, and GA-FA-CSLNs) at two concentrations (0.3, 0.6 μg/mL) for 48 h. Two concentrations (5, 10 μg/mL) of cisplatin were used as the positive control group. The cells were collected and treated with trypsin and then centrifuged at 1000 rpm for 5 min and fixed with 75% ethanol at −20 °C for 24 h. After that, the cells were centrifuged at 800 rpm for 5 min and washed with PBS; this process was repeated twice to remove the ethanol. Afterwards, 150 μL PI solution containing 50 μg/mL PI and 100 μg/mL RNase A was added followed by incubation at 4 °C for 30 min. The cell cycle was evaluated with flow cytometry (CytoFLEX).

### 4.8. Cellular Uptake and Competition Suppression Assay

Fluorescence microscopy was used to investigate the qualitative uptake of SLNs modified with C6 by HepG2 cells. The cell uptake experiment consisted of five groups, C6, C6-CSLNs, GA-C6-CSLNs, FA-C6-CSLNs, and GA-FA-C6-CSLNs, and two concentrations of C6 (40 μg/mL, 80 μg/mL) for each group. The specific procedure was as follows: HepG2 cells (1 × 10^5^/well) at logarithmic growth stage were incubated in 24-well cell culture plates and cultured for 24 h. In the cell uptake experiments, 1 mL of SLNs was added to the plates and cultured at 3 °C for 4 h; however, in the competition inhibition test group, serum-free DMEM medium containing FA ligands (0.5 mg/mL) was added for incubation for 2 h, then supernatant was discarded, followed by the addition of 1 mL of FA-C6-CSLNs or GA-FA-C6-CSLNs of culture for 2 h. Afterwards, the cells were fixed with 75% ethanol solution for 10 min, stained with 150 μL of 200 ng/mL DAPI for 15 min, and then washed three times with PBS. Finally, cells were observed under fluorescence microscopy.

### 4.9. In Vivo Efficacy Evaluation

Tumor-bearing mice were prepared by inoculating HepG2 cell suspension (100 µL, 1 × 10^7^/mL) into each axillary fossa of BALB/c nude mice. Animals were bred in an SPF facility and had free access to food and water. When the tumor diameter reached 8 mm × 8 mm, the mice were divided into six groups, with six mice in each group. The groups were treated tail intravenously with physiological saline, CSLNs, GA-CSLNs, FA-CSLNs, and GA-FA-CSLNs (375 μg/kg), respectively; the positive control group was intraperitoneally injected with cisplatin at 2 mg/kg, administered every other day for a total of 7 times. The body weight was measured, and the tumor length and diameter were recorded each time. Then, the mice were sacrificed, and the local tumors were removed and weighed carefully. The tumor inhibition rate (IR) was calculated with the following equation:

IR(%)=(Wcontrol−WSLNs)/Wcontrol×100%,

where Wcontrol is the average weight of the control group and WSLNs is the average weight of the experimental group.

### 4.10. Statistical Analysis

All statistical data were obtained from at least three parallel experiments, and the data are represented as mean ± SD. Tumor inhibition rate data were analyzed by one-way ANOVA with Tukey’s multiple comparison test using the software GraphPad Prism v7.00.

## 5. Conclusions

In this paper, novel CTD-loaded solid lipid nanoparticles modified by GA and FA were constructed with the aim of increasing antitumor efficiency and liver-targeting activity.

Four CTD-loaded SLNs prepared under the optimum conditions had a clear and transparent appearance that was accompanied by bluish or yellowish opalescence and an average particle size of less than 120 nm, matching the expected particle size requirements of liver targeting. The entrapment efficiency was greater than 95%, and the drug loading was approximately 0.35%. The preparation had no hemolysis reaction, laying the foundation for intravenous administration in vivo.

In vitro cell experiments showed that FA-CSLNs and GA-FA-CSLNs had stronger cytotoxicity and could reduce the migration rate of HepG2 cells compared with GA-CSLNs and CSLNs, and the toxicity of GA-FA-CSLNs on normal liver cells (L-02) was significantly lower than that of other groups. Furthermore, cells in the FA-CSLNs groups had the highest rate of apoptosis. The inhibition mechanism against HepG2 cells was mainly through arresting the cell cycle at the S phase and G2/M phase. The results of competition inhibition assays indicated that GA and FA ligands had targeting effects on HepG2 cells, and this result was corroborated well with the in vivo efficacy evaluation experiment: SLNs modified with double ligands had better in vivo antitumor efficacy. These results support our hypothesis that solid lipid nanoparticles containing FA and GA ligands represent a potential targeted therapy drug carrier for hepatocellular carcinoma.

## Figures and Tables

**Table 1:** Characteristics of CSLNs (x ± s, *n* = 3).

Samples	Particle (Sizenm)	PDI	Zeta-Potential (mV)	EE(%)	DL(%)
CSLNs	75.42 ± 1.14	0.191 ± 0.013	−9.66 ± 0.18	96.75 ± 0.26	0.37 ± 0.02
GA-CSLNs	105.43 ± 2.75	0.495 ± 0.011	−10.80 ± 0.16	96.31 ± 0.26	0.35 ± 0.03
FA-CSLNs	75.91 ± 0.71	0.202 ± 0.007	−10.98 ± 0.87	96.95 ± 0.39	0.38 ± 0.02
GA-FA-CSLNs	78.01 ± 1.03	0.487 ± 0.015	−10.16 ± 0.11	95.98 ± 0.21	0.32 ± 0.04

**Table 2:** Hemolysis experiment design scheme.

Number	Sample (mL)	0.9% NaCl Solution (mL)	2% Red Blood Cell Suspension (mL)	Deionized Water (mL)
1	0.2	2.3	2.5	0
2	0.3	2.2	2.5	0
3	0.4	2.1	2.5	0
4	0.5	2.0	2.5	0
5	0.6	1.9	2.5	0
6	0.7	1.8	2.5	0
7	0.0	2.5	2.5	0
8	0.0	0.0	2.5	2.5
9	0.7	4.3	0.0	0

## Data Availability

The data presented in this study are available on request from the corresponding author.

## Sample Availability

Not available.
